# Feasibility of a Reinforcement Learning–Enabled Digital Health Intervention to Promote Mammograms: Retrospective, Single-Arm, Observational Study

**DOI:** 10.2196/42343

**Published:** 2022-11-28

**Authors:** Amy Bucher, E Susanne Blazek, Ashley B West

**Affiliations:** 1 Lirio Knoxville, TN United States

**Keywords:** artificial intelligence, reinforcement learning, feasibility studies, mammograms, nudging, behavioral intervention, digital health, email, health equity, cancer screening

## Abstract

**Background:**

Preventive screenings such as mammograms promote health and detect disease. However, mammogram attendance lags clinical guidelines, with roughly one-quarter of women not completing their recommended mammograms. A scalable digital health intervention leveraging behavioral science and reinforcement learning and delivered via email was implemented in a US health system to promote uptake of recommended mammograms among patients who were 1 or more years overdue for the screening (ie, 2 or more years from last mammogram).

**Objective:**

The aim of this study was to establish the feasibility of a reinforcement learning–enabled mammography digital health intervention delivered via email. The research aims included understanding the intervention’s reach and ability to elicit behavioral outcomes of scheduling and attending mammograms, as well as understanding reach and behavioral outcomes for women of different ages, races, educational attainment levels, and household incomes.

**Methods:**

The digital health intervention was implemented in a large Catholic health system in the Midwestern United States and targeted the system’s existing patients who had not received a recommended mammogram in 2 or more years. From August 2020 to July 2022, 139,164 eligible women received behavioral science–based email messages assembled and delivered by a reinforcement learning model to encourage clinically recommended mammograms. Target outcome behaviors included scheduling and ultimately attending the mammogram appointment.

**Results:**

In total, 139,164 women received at least one intervention email during the study period, and 81.52% engaged with at least one email. Deliverability of emails exceeded 98%. Among message recipients, 24.99% scheduled mammograms and 22.02% attended mammograms (88.08% attendance rate among women who scheduled appointments). Results indicate no practical differences in the frequency at which people engage with the intervention or take action following a message based on their age, race, educational attainment, or household income, suggesting the intervention may equitably drive mammography across diverse populations.

**Conclusions:**

The reinforcement learning–enabled email intervention is feasible to implement in a health system to engage patients who are overdue for their mammograms to schedule and attend a recommended screening. In this feasibility study, the intervention was associated with scheduling and attending mammograms for patients who were significantly overdue for recommended screening. Moreover, the intervention showed proportionate reach across demographic subpopulations. This suggests that the intervention may be effective at engaging patients of many different backgrounds who are overdue for screening. Future research will establish the effectiveness of this type of intervention compared to typical health system outreach to patients who have not had recommended screenings as well as identify ways to enhance its reach and impact.

## Introduction

In the United States, breast cancer is the second most common type of cancer among women [1]. Globally, it represented 11.7% of all new cancer cases in 2020, accounting for 1 in 4 cancer diagnoses among women [2]. Mammograms are a valuable tool in detecting breast cancer early, when treatment options may be less invasive, intensive [3], and costly [4], and are associated with lower frequencies of advanced and fatal breast cancer [5]. Many patients do not generally adhere to national guidelines on the frequency and timing of preventative care [6]. Adherence to recommended mammogram screenings is no different. According to the Behavioral Risk Factor Surveillance System’s 2020 US data, only 78.2% of women between the ages of 50 and 74 years had a mammogram in the past 2 years [7]. The COVID-19 pandemic further exacerbated the gap between recommended and attended screenings, with mammogram rates falling dramatically in April 2020 compared to April 2019 [8]. While rates have since rebounded to close to prepandemic levels, it is estimated that it could take as long as 22 weeks to clear the backlog of delayed mammograms [9]. While there is some debate over appropriate mammogram usage given drawbacks associated with overdiagnosis [10], the gap between recommended and actual screening behavior is likely to persist regardless of adjustments to the recommendations, suggesting the need for behavioral interventions targeting those who remain overdue for mammograms.

Evidence suggests that such behavioral interventions are most effective when they address a comprehensive set of barriers to performing a health behavior [11-13], apply different behavior change ingredients to overcome each barrier [12-14], and personalize these ingredients to each person as their barriers dynamically change over time [15,16]. Tailoring digital health messages overcomes person-specific barriers [15,17], facilitates the behavior [15,17], and, most notably, improves health outcomes [14-16]. However, personalizing behavior change ingredients to people’s changing barriers over time involves reassessing barriers [18], which is labor intensive, costly, and not scalable [13]. Sophisticated technologies, such as artificial intelligence (AI), offer promise to overcome some of these limitations [19], but these are not yet widely used.

It is important to note that while digital health presents a promising means to deliver interventions at scale, not all people are equally likely to use technology for health-related purposes. For example, Black and Hispanic people engage less in digital health than their White counterparts [20], and many people in both rural and urban areas lack broadband internet access to support more data-intensive applications [21]. Email remains a relatively accessible modality to deliver behavioral interventions, being widely used across racial and ethnic groups [20] and requiring much less data than an app to access on a computer or mobile device. Emails are also a typical method for health systems to communicate with patients, meaning little additional technological support is required to use them in that environment, and patients are familiar with this type of digital interaction with their health system.

The purpose of this study is to assess the feasibility of developing and implementing a digital health intervention incorporating reinforcement learning, a type of AI, to personalize email content in order to increase mammography scheduling and attendance among patients of a large health system who are significantly overdue for their recommended screenings. This retrospective, single-arm, observational study explores the reach of the intervention within the patient population, outcomes related to engagement, and outcomes related to the target behaviors of scheduling and attending mammograms.

## Methods

### Background

This study had several purposes. First, we sought to establish the feasibility of implementing a behavioral science–informed, reinforcement learning–powered digital health intervention intended to increase the scheduling of and attendance at mammograms within a health system. Next, we wanted to understand the reach of such an intervention within the patient population. Finally, the study was intended to identify the behavioral outcomes of mammogram scheduling and attendance associated with use of the intervention.

### Ethical Considerations

Solutions IRB, a private institutional review board accredited by the Association for the Accreditation of Human Research Protections Programs, approved analyses of deidentified, aggregated derived data with a waiver of informed consent (study ID: 2021/05/28). Study data were deidentified and anonymous.

### Setting and Participants

The intervention was implemented in a large Catholic health system in the Midwestern United States. The implementation focused on patients who were overdue for, and eligible to schedule, a recommended mammogram. Patients were eligible for the intervention if they were female, between 49.5 and 74 years of age, had not had a mammogram in the past 24 months, were subscribed to health system communications, and had a valid email address on file. Patients were excluded if they had a future mammogram scheduled, had a history within the last 12 months of a breast cancer diagnosis or associated surgery, had health maintenance modifiers excluding them from outreach, or indicated participation in hospice, palliative care, or long-term nursing home care.

### Data Collection and Rolling Eligibility

At intervention launch, the health system provided a population-level historical data file of all patients from their Epic electronic medical record system to facilitate the establishment of eligibility criteria and set up data integration. This data file included the email addresses for which the system has permission to contact patients about health-related matters. Then, during the study period, the system sent daily data file updates with information on whether patients had scheduled or attended a mammogram (behavioral outcome), as well as changes to age, health status, or other variables affecting eligibility for communication. Patients whose data rendered them newly eligible or ineligible for the intervention were added or removed to the distribution list accordingly. Eligible individuals who did not schedule and attend a mammogram and who did not unsubscribe continued to receive communications for the duration of the study period. Eligible individuals received up to 40 emails during the 2-year study period.

Data for this study were collected between August 27, 2020, and July 12, 2022.

### Intervention

Precision Nudging for mammography is an English-language messaging intervention designed to influence the target behaviors of scheduling and attending a mammogram. The messages are designed to address specific determinants of completing a mammogram, identified through a combination of literature review and primary research with health systems. A sample of these determinants can be found in Textbox 1. Those determinants are then organized using an intervention mapping process [22] that links barriers and facilitators to evidence-based behavior change techniques (BCTs) [23]. The BCTs are operationalized into a set of message components, such as subject lines, body copy paragraphs, and visual illustrations, that form a content library [24]. Interrater agreement of the content (ie, subject lines and body content) was assessed by two trained coders [25] to ensure that each component accurately operationalized the intended BCT; agreement exceeded the acceptability threshold of κ=0.80.

**Figure 1:**
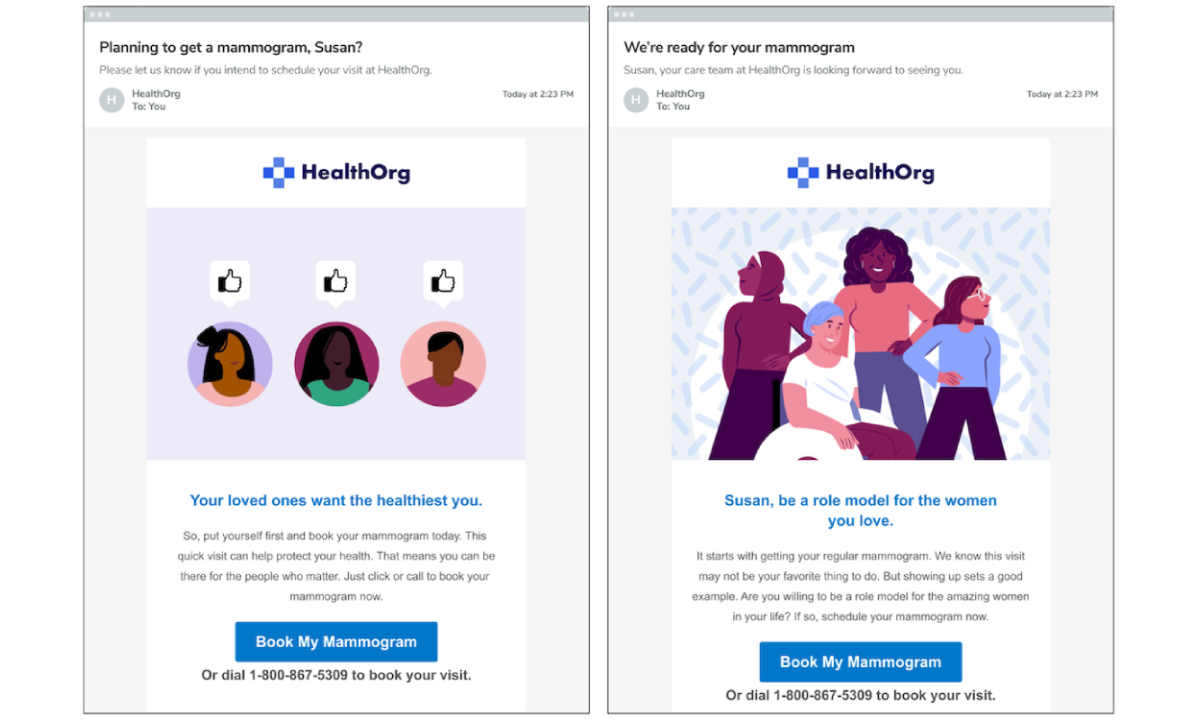
Sample assembled messages from the Precision Nudging mammography intervention.

A behavioral reinforcement learning (BRL) algorithm [26-29] then selected components to compile into a complete message based on recipient characteristics. A total of 468 email combinations were possible using the components used in this implementation. Over time, the BRL algorithm optimized message composition based on recipients’ past behavioral responses (ie, opening messages, clicking calls to action, and scheduling and attending mammograms) by selecting components that maximize the probability that the recipient will complete the target behaviors. All emails were white labeled so that they appeared to come from the health system. Figure 1 shows some sample assembled messages.

Approximately 30 days prior to launch, we conducted an IP warming exercise intended to establish a reputation for the IP address used to send intervention emails. This minimizes the likelihood that intervention emails will be flagged as spam by the most common email providers, including Gmail and Yahoo.

**Figure 2:**
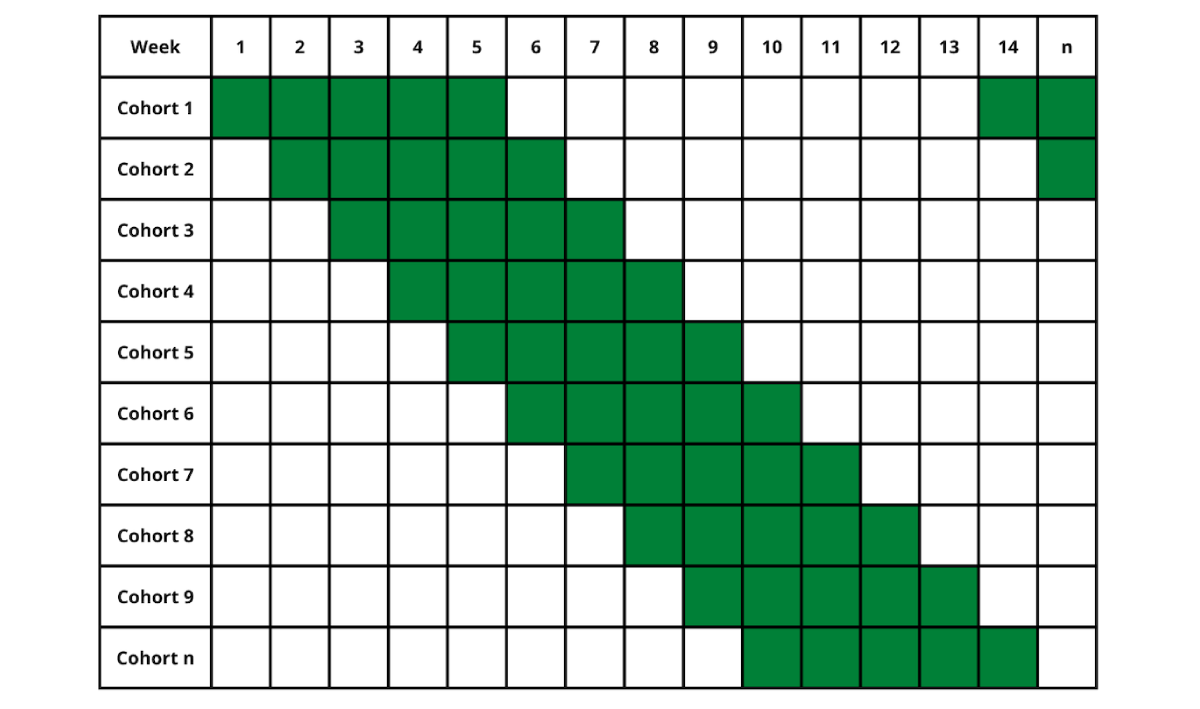
An example of the messaging cadence by cohort showing alternating pulses of one message per week for 5 weeks and an 8-week pause.

In order to avoid creating excess demand on the health system, eligible women were randomly assigned to cohorts of approximately 2000 people; the intervention start date was staggered across cohorts. Intervention communications were sent out once per week on Tuesday mornings via a third-party email vendor. Each cohort received one message per week for 5 weeks, with an 8-week pause, and then another pulse of one message per week for 5 weeks. This message patterning was designed to balance intervention exposure and potential notification fatigue. This pattern continued until women scheduled a mammogram, unsubscribed from the intervention, or otherwise became ineligible for continued communication. Figure 2 shows the communication patterns incorporating both cohorts and message timing.

The calls to action to schedule a mammogram were based on the location where each eligible patient received care according to the eligibility data file provided by the health system. For all care locations, patients were provided the appropriate scheduling phone number. Patients with an established patient portal account who received care at a location with online scheduling enabled also received a link to schedule in the portal.

**A sample of the determinants for mammogram scheduling and attendance incorporated into the development of the Precision Nudging intervention.**

**Intrapersonal barriers**

- Low perceived risk
- Fear of diagnosis

**Social context barriers**

- Lack of social support
- Social norms around mammograms

**Environmental context barriers**

- Cost
- Scheduling and wait times

### Outcomes

We assessed the feasibility of the Precision Nudging intervention by investigating engagement with emails, measured via open rates and clicks on the call to action, and behavioral outcomes, measured as mammograms scheduled and attended. Clicking the call to action was not a prerequisite for successfully scheduling or attending a mammogram, as patients had options to either call for an appointment or self-navigate to their patient portal for online scheduling. We also examined the demographic characteristics of the women who received, engaged with, and took action following a Precision Nudging communication.

### Data Analysis

Data were analyzed using the Python programming language. Univariate statistics were used to understand who was reached with the intervention and who responded by scheduling or attending mammograms. Chi-square goodness-of-fit tests were used to understand whether any demographic groups were more likely than others to engage or take action following an intervention message.

## Results

### Email Deliverability

During the study period, a total of 2,761,270 messages were sent. Overall, 98.91% of emails sent were successfully delivered to a total of 139,164 women (ie, reached). A total of 32.35% of emails were opened at least once. Over the study period, a total of 14,625 women (10.51%) unsubscribed from the intervention messaging.

### Demographic Reach

Of the 139,164 women reached with the intervention, the majority (n=121,909, 87.60%) were Caucasian, with the next largest racial group being Black (n=11,879, 8.54%). The most common level of educational attainment was completion of high school (n=61,001, 43.83%), and the majority of message recipients had a household income level under US $100,000 (n=101,164, 72.69%). The mean age of message recipients was 62.13 (SD 7.23) years. One person older than 80 years received an intervention message; this was in error, as eligibility to receive mammography outreach requires patients be 74 years of age or younger. The sample characteristics are summarized in Table 1.

**Table 1:** Summary of the demographic characteristics of the women receiving intervention messages, with engagement and behavioral responses.

Characteristics			Reached (N=139,164), n (%)		Opened message (n=113,452), n (%)^a^		Clicked call to action (n=15,636), n (%)^a^		Scheduled mammogram (n=34,780), n (%)^a^		Attended mammogram (n=30,637), n (%)^a^
**Age (years)**											
	50-59	58,439 (41.99)		48,240 (82.55)		7825 (13.39)		15,242 (26.08)		13,368 (22.88)	
	60-69	56,047 (40.27)		45,671 (81.49)		5938 (10.59)		14,489 (25.85)		12,801 (22.84)	
	70-79	24,677 (17.73)		19,541 (79.19)		1873 (7.59)		5049 (20.46)		4468 (18.11)	
	80-89	1 (0)		0 (0)		0 (0)		0 (0)		0 (0)	
**Race**											
	Caucasian	121,909 (87.60)		99,649 (81.74)		13,272 (10.89)		30,104 (24.69)		26,670 (21.88)	
	Black	11,879 (8.54)		9370 (78.88)		1773 (14.92)		3431 (28.89)		2848 (23.98)	
	Asian	659 (0.47)		554 (84.07)		90 (13.66)		230 (34.90)		208 (31.56)	
	Two or more races	187 (0.13)		137 (73.26)		17 (9.09)		40 (21.39)		36 (19.25)	
	Other	4530 (3.25)		3742 (82.60)		484 (10.68)		975 (21.52)		875 (19.32)	
**Educational attainment**											
	Completed high school	56,767 (40.79)		45,665 (80.44)		6158 (10.85)		15,889 (26.99)		13,920 (24.52)	
	Completed vocational or technical training	1585 (1.14)		1281 (80.82)		177 (11.17)		425 (26.81)		378 (23.85)	
	Completed college	44,054 (31.66)		36,389 (82.60)		5232 (11.88)		12,137 (27.55)		10,776 (24.46)	
	Completed graduate school	17,747 (12.75)		15,217 (85.74)		2115 (11.92)		4397 (24.78)		3924 (22.11)	
	Unknown	18,901 (13.58)		14,838 (78.50)		1974 (10.44)		5218 (27.60)		4551 (24.08)	
**Household income level (US $)**											
	<40,000	35,306 (25.37)		27,129 (76.84)		3564 (10.09)		9176 (25.99)		7966 (22.56)	
	40,000-69,999	30,374 (21.83)		24,312 (80.04)		3293 (10.84)		7870 (25.91)		6962 (22.92)	
	70,000-99,999	35,964 (25.84)		29,944 (83.26)		4232 (11.77)		8989 (24.99)		7907 (21.99)	
	≥100,000	32,608 (23.43)		28,275 (86.71)		4032 (12.37)		7467 (22.90)		6678 (20.48)	
	Unknown	4912 (3.53)		3792 (77.19)		515 (10.48)		1278 (26.02)		1124 (22.88)	

^a^Percentages in these columns are based on values in the “Reached” column.

### Engagement With Messages

Overall, of the 139,164 people who received an intervention email, 113,452 (81.52%) opened at least one message. A total of 15,636 people (11.24%) clicked the call to action in at least one of the messages (ie, “clicks”). Engagement by sample characteristics can be found in Table 1.

For those who opened an email, it took an average of 4.46 (SD 5.87) emails for them to do so. For those who clicked an email, it took opening of an average of 4.26 (SD 4.49) emails first; the data do not indicate how many emails were opened prior to people booking a mammogram by phone or via the patient portal without clicking a call to action. Time delays in the scheduling and attendance data prevent calculating the average number of messages prior to behavioral engagement.

### Behavioral Outcomes

Among the 139,164 people messaged, 34,780 people (24.99%) scheduled an appointment for a mammogram. At the time of data analysis, 30,637 people (22.02% of the total; 88.09% of those who scheduled) had attended a mammogram. Behavioral response by sample characteristics can be found in Table 1.

### Proportionate Engagement and Behavioral Outcomes Across Demographic Subgroups

An important goal in digital health intervention development and research is the achievement of health equity, reached when every person has the opportunity to “attain his or her full health potential” [30]. Statistical methods for analyzing health equity—or equivalent outcomes between groups—largely stem from clinical trial research and tend to focus on comparisons between two groups (ie, two means, two proportions, etc) [31].

One approach to showing equivalence is to carry out a chi-square goodness-of-fit test based on the null hypothesis of no treatment difference [32]. Chi-square goodness-of-fit tests were used to analyze whether any patient subsamples were more likely than others to engage with the intervention emails (ie, opened and clicked). Due to giant sample sizes, all the chi-square goodness-of-fit tests were significant at *P*<.001—with the exception of the chi-square goodness-of-fit test comparing email engagement between race subgroups, which was significant at *P*=.008—obscuring the fact that the expected engagement resembled the observed engagement across demographic subgroups. In samples of this size, the *P* values quickly approach zero [33]. Unlike in clinical trials, where insufficient sample sizes, insensitive outcome measures, or insensitive analyses unduly threaten nonsignificant results [32], this research is challenged by the giant sample size. Basing conclusions on the *P* values of the chi-square goodness-of-fit tests might suggest that there are important statistical differences in engagement between population subgroups, when in fact the results indicate little to no practical difference.

For example, women across age groups demonstrated practically similar levels of engagement with the emails and close-to-expected values from the baseline established by the percentage of women in each group reached. In total, 82.55% of the 58,439 women reached who were aged 50 to 59 years (n=48,240) opened an email, compared to the expected baseline of 81.60%. Even closer to the baseline, 81.49% of the 56,047 women reached who were aged 60 to 69 years (n=45,671) opened an email. Slightly further from the baseline were women aged 70 to 79 years, of whom 79.19% out of 24,677 women reached (n=19,541) opened an email.

Chi-square goodness-of-fit tests were also used to analyze whether any patient subsamples were more likely than others to schedule or attend a mammogram following receipt of the intervention emails. Similar to the tests conducted for email engagement, due to giant sample sizes, all the chi-square goodness-of-fit tests for behavioral outcomes were significant at *P*<.001. Again, the large sample sizes obscure the fact that the expected engagement resembled the observed engagement across demographic subgroups.

For example, women across income levels demonstrated practically similar levels of behavioral outcomes and close-to-expected values based on the baseline. Out of 35,306 women reached who were making less than US $40,000 per year, 22.56% (n=7966) attended a mammogram, compared to the expected baseline of 25.37%. Out of 30,374 women reached who were making US $40,000 to US $69,999 per year, 22.92% (n=6962) attended a mammogram; also, 21.99% of the 35,964 women reached making US $70,000 to US $99,999 per year (n=7907) attended a mammogram. Slightly further from the baseline were the 32,608 women making more than US $100,000 per year, of which 20.48% (n=6678) attended a mammogram. Out of 4912 women with unknown income, 22.88% (n=1124) attended a mammogram.

**Figure 3:**
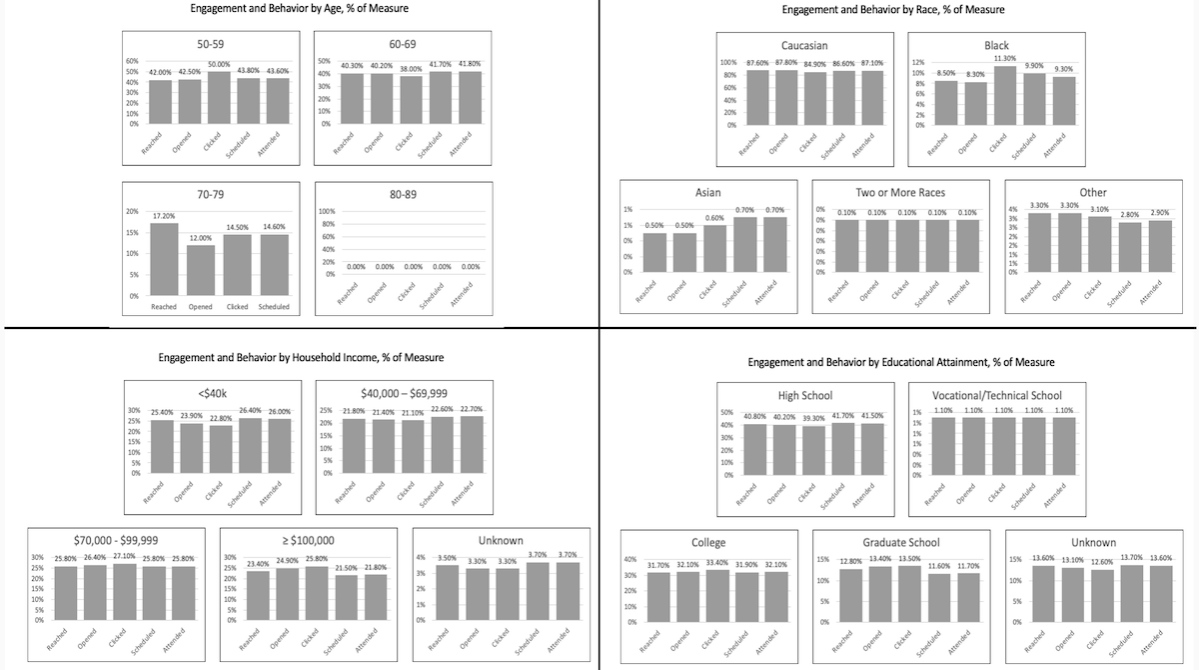
Bar graphs showing the proportion of each demographic subgroup who engaged and took action after receiving an intervention. Relatively small changes down the funnel from reached to opened to clicked to scheduled to attended suggest, from a practical perspective, proportional response to the intervention for people in that group.

An alternative approach to a chi-square goodness-of-fit test based on the null hypothesis of no treatment difference is to preselect a value for the treatment difference that is of practical importance [32,34]. This value should be chosen a priori such that proportions can be considered equivalent if their observed differences do not exceed it. Figure 3 shows the proportion of each demographic subgroup for each measure along the behavioral funnel. We did not choose an a priori value for the treatment difference because of the novel nature of the reinforcement learning–enabled digital health intervention under study. Instead, these exploratory analyses seek to complement the chi-square goodness-of-fit tests and to qualitatively ascertain equitable reach by demonstrating that the proportion of each demographic subgroup remains close to the same for each measure along the behavioral funnel.

For example, 87.60% of the reached population consisted of Caucasian people, so equivalent outcomes would require that close to 87.60% each of the populations who opened at least one message, clicked the call to action, scheduled a mammogram, and attended a mammogram should consist of Caucasian people. For the population who opened at least one message, the proportion of Caucasian people was slightly higher than the expected 87.60%. For the populations who clicked the call to action, scheduled a mammogram, and attended a mammogram, the proportion of Caucasian people was slightly lower than the expected 87.60%.

## Discussion

### Reach and Behavioral Outcomes

This study explored the feasibility of developing and implementing Precision Nudging (ie, a tailored messaging intervention delivered through a BRL algorithm) to promote mammograms to eligible patients of a large health system. Overall, of the 139,164 people who received an intervention email, 113,452 (81.52%) opened at least one message and 15,636 (11.24%) clicked a call to action. A total of 34,780 people (24.99%) scheduled a mammogram, and 30,637 people (22.02% of the total; 88.08% of those who scheduled a mammogram) attended a mammogram. The results support similar health-related pilot studies [35-39] and demonstrate that a reinforcement learning–enabled digital health intervention is capable of reaching women overdue for recommended screenings and prompting behavioral responses, such as scheduling and attending mammograms. The results also demonstrate that engagement and behavioral response are proportional within demographic subgroups of race, age, educational attainment, and household income level.

The intervention used in this study was specifically designed to address a broad range of behavioral determinants, including those more common among underserved groups, like racial and ethnic minorities, and those experiencing poor social determinants of health [24]. A test of whether that approach was successful is assessing whether engagement and behavioral responses from members of those groups are at levels equal to or greater than responses from people who are Caucasian or of higher socioeconomic status. Within the population who was eligible to receive intervention messaging, we saw no practical differences [40] in the frequency at which people engaged with the intervention or took action following a message based on their age, race, educational attainment, or household income. This has implications for the ability of this type of intervention to support health equity in being able to communicate with, and overcome barriers to, preventive care across population subgroups, including many that are historically underserved by digital health, such as non-White people and people of lower educational and income levels. Importantly, this study suggests that such equitable outreach can be done at scale by leveraging email, reinforcement learning, and behavioral science.

That said, we do see differences in the baselines by which people of demographic subgroups were reached by the intervention. For example, 17.73% of the sample were women aged 70 to 79 years, compared to 41.99% who were women aged 50 to 59 years and 40.27% who were women aged 60 to 69 years. While it is likely this is partly due to the fact that eligibility criteria for the intervention was cut off at 74 years of age, curtailing the number of potentially eligible women, it is worth investigating alternative channels to ensure that people who are not frequent email users receive prompts about recommended health behaviors.

It is important to note that the patients included in the feasibility pilot were considered less engaged with their health care by nature of being overdue for their recommended mammograms without a future appointment scheduled. Highly activated patients tend to be compliant with health recommendations, including breast cancer screening [41]. Relatedly, in this sample, 88.08% of women who scheduled a mammogram went on to attend it, suggesting a no-show rate as high as 11.92%. This is higher than the no-show rates found in other research studies looking at a general population (ie, 6.20%) [42]. It seems likely that expanding this intervention to a more heterogeneously engaged sample (ie, women within 6 to 24 months of their last mammogram) may yield higher mammogram scheduling and completion rates.

### Implications and Future Directions

Having established the feasibility of this digital health intervention to improve uptake of mammograms in a health system, the most obvious and urgent next steps are to understand causal effects. It is important to understand whether this intervention improves mammogram uptake compared to standard of care or alternatives, such as a static reminder message. This research may be accomplished via a randomized controlled trial or quasi-experimental implementation (eg, comparing synchronous mammogram rates between two similar health systems or markets where one uses the intervention and the other does not). It also may be fruitful to look at historical screening behaviors among the patients eligible for the intervention—or a yoked sample of similar patients—to establish the incremental influence of Precision Nudging on mammography behaviors.

Future research should investigate the economics of a behavioral intervention such as this one to increase mammography uptake in a health system. Given the potential of mammography to detect breast cancers at an earlier stage where treatment is less costly, widespread implementation of this sort of intervention may yield observable return on investment at the health system level over time, especially if used with unengaged patients who may have historically skipped recommended screenings and checkups. Understanding the economic impact of mammography interventions will help health systems determine whether and when to implement such programs as part of their prevention and disease management portfolios. As this research will necessarily account for cost savings associated with early detection as well as expenses associated with false positives, it may also help to clarify the costs and benefits of annual mammograms, in general, and for specific demographic groups.

Another promising area of future study is the use of behavioral interventions to improve operational efficiency in health systems. A potential drawback to patient-directed behavioral interventions is that they may increase provider workload. The Precision Nudging intervention was designed to have limited impact on clinician workflow. Patient data were automatically captured from the medical record without additional steps from providers, and communication timing and frequency were considered in terms of provider capacity. The intervention also accommodates message throttling to help mitigate excess demand on screening centers. We believe that interventions that help close the loop within the health system so that patients complete recommended behaviors in a timely manner may actually create operational efficiencies by smoothing demand for mammograms and other screenings and make productive use of existing patient data to support engagement with recommended care. It may also have the benefit of making it clear to providers which patients do not have or use email and may require high-touch outreach, so that those channels can be used appropriately. It would be worth quantifying whether monitoring and adjustment of outreach smooths mammography schedules, maximizing throughput without creating additional stress on providers. This could include both increasing mammogram appointments at slow times of the day, week, month, or year, as well as shifting mammography demand subsequent to campaigns such as Breast Cancer Awareness Month in October [43] to times where capacity is greater.

Another lens to understand how the intervention impacts patient behaviors is through patient experience research. Especially given that the population reached in this feasibility study was not proactively engaged in scheduling their recommended mammograms, there is value in a qualitative understanding of their response to the email communications and whether they perceived them as different or more compelling than typical health system communications. We hope to study patient perception of Precision Nudging as well as perception of the communications to better understand the intervention’s effects and continually improve its acceptability and effectiveness.

Finally, there is opportunity beyond patient experience research to investigate improvements to the intervention itself. This may include advances to the reinforcement learning platform that assembles messages based on patient behavioral responses, enhancements to the library content to address barriers more effectively or to accommodate emerging barriers, or expansion to other channels, such as text message or chatbot, to better engage the full patient population. Although the feasibility study shows promising reach and engagement across patient subgroups, future research should focus on ensuring equitable access and support for preventive care among groups with historical experience of structural inequalities [44]. This will require engaging members of those groups to understand their barriers to action and partnering to co-design solutions [24]. Ensuring equity will also require that the data used to train interventions like the one under study, which is driven by reinforcement learning, are representative of the populations at large and that the benefits conferred are available to all [45].

### Limitations

This feasibility study offers real-world pilot results, while laying the groundwork for further investigation. First, and most obviously, while this study demonstrates that a BRL-powered email-based behavioral intervention is feasible to deliver a behavioral intervention for mammograms, its efficacy in achieving behavioral results can be better understood through a randomized controlled trial or other experimental methods in future work. The hypothesis that the personalization enabled by BRL enhances outcomes relative to a standard nonpersonalized health system messaging campaign should be rigorously tested.

In terms of better demonstrating equitable outcomes, a major limitation to this study came from the lack of an a priori value for the treatment difference that would be of practical importance. Given the giant sample sizes in studies like this, alternative methods for establishing equivalence need to be employed. Future research should suggest and test a priori values of true treatment differences between population subgroups.

This work was also confined to a single health system whose patients are geographically concentrated in the Midwestern United States. Moreover, the system is a mission-driven Catholic health care organization. Although this does not have bearing on recommendations around breast cancer screening, the health system’s Catholic identity may attract a patient population who differs from the general US patient population. Future research should examine the success of this mammography intervention in other health systems to establish the generalizability of the results.

The unsubscribe rate for intervention emails over the 2 years of study was 10.5%. Despite being generally much higher than the 2022 average for health care services marketing emails [46], it is difficult to draw direct comparisons between this novel reinforcement learning–driven digital health intervention and other digital communications interventions. In this case, it is reasonable to assume that a large proportion of those who unsubscribed were women who had attended their mammograms, as they were not informed that they would no longer receive intervention emails once they had scheduled or attended their mammograms. Other women might have been induced to feel annoyed or guilty by the ongoing messages. Thus, a limitation involved not exploring the demographics, engagement, and behavioral outcomes of those who unsubscribed, to better understand their motivations for doing so, and, ultimately, improve the intervention to reduce the unsubscribe rate.

Finally, the time period during which the feasibility study was conducted coincided with the resumption of preventive care, such as mammograms, during the COVID-19 pandemic, which may have artificially influenced mammography behaviors among the patient population. Continued monitoring of the intervention’s outcomes over time should provide clarity as to its performance in times of reduced demand for screenings.

### Conclusions

This retrospective, single-arm, observational study suggests that a reinforcement learning–enabled email intervention can be used in a health system to engage patients who are significantly overdue for their mammograms to schedule and attend a recommended screening. In this feasibility investigation, the intervention was associated with scheduling and attending mammograms for patients who were significantly overdue for recommended screening. Moreover, the intervention showed proportionate reach across demographic subpopulations. This suggests that the intervention may be effective at engaging overdue patients of many different backgrounds. In a time where many patients are behind on preventative screenings, with potentially life-altering results, and where many health care organizations are eager to manage costs and deliver quality care, interventions that engage the most disengaged patients are a vital tool to improve outcomes. These interventions will be successful to the extent they can be delivered in a low-cost and scalable fashion, offer flexibility to systems and providers to support established workflows, and concretely help patients overcome the barriers that have kept them from recommended care.

## Abbreviations

AI: artificial intelligence
BCT: behavior change technique
BRL: behavioral reinforcement learning

## Data Availability

The data sets generated and analyzed during this study are not publicly available due to contractual agreements between Lirio and the health system where the intervention was implemented. Derived data used in the analyses are available from the corresponding author upon reasonable request.
